# *Beronaphaenops paphlagonicus*, a new anophthalmous genus and species of Trechini (Coleoptera, Carabidae) from Turkey

**DOI:** 10.3897/zookeys.255.4173

**Published:** 2012-12-27

**Authors:** Borislav V. Guéorguiev

**Affiliations:** 1 National Museum of Natural History, 1 Blvd. Tzar Osvoboditel, 1000 Sofia, Bulgaria

**Keywords:** Coleoptera, Carabidae, Trechini, taxonomy, new genus, new species, Turkey

## Abstract

*Beronaphaenops* **gen. n.** *paphlagonicus* **sp. n.**, a new remarkable, eyeless species of Trechini is described from Asian Turkey (type locality: cave Eşek Çukuru Mağarası 2, Milli Park Küre Dağlari, Pinarbasi District, Kastamonu Province). This specialized, troglobite species is characterized by a very peculiar combination of features, including several autapotypic features: mentum tooth large, long and porrect, at distal position reaching or slightly exceeding the level of epilobes, rather slanting ventrally, deeply bifid at the tip; short and fragile paraglossae, hardly surpassing the anterior margin of ligula; absence of posterolateral setae of the pronotum; absence of posterior discal pore in elytral stria 3; apical stylomere shortened, with basal part unusually broadened. The systematic position of the genus amongst the trechine beetles from the peri-Pontic area is discussed. A key to the Anatolian genera of the tribe is prepared.

## Introduction

Up to now 19 species of blind Trechini have been recorded from Anatolia, belonging to 6 genera (Vigna Taglianti 1980; Casale and Vigna Taglianti 1999; Moravec et al. 2003). In consideration of the size of this landmass, its relief and suitable habitats it includes, this is astonishingly a small number compared to the numerous species and genera documented for the faunas of two next-door regions, the Balkan Peninsula and the Caucasus (Belousov 1998; Moravec et al. 2003; Quéinnec 2008; Lohaj and Lakota 2010; Casale et al. 2012). Maybe this fact is a consequence of still unexplored underground communities in the region considered. But, an ecological explanation connected with a specific geological history seems more probable. Casale and Vigna Taglianti (1999: 303) supposed the unfavorable pressure of former climatic conditions in Anatolia has been “*lower in modifying markedly the adaptive features of carabids to the subterranean environment, being the cave super-specialized taxa almost absent*”, in contrast to that in other Mediterranean areas.

It is worth noting that the blind Trechini from the area in question belong to three phyletic lines (Jeannel 1928; Casale and Laneyrie 1983; Casale and Vigna Taglianti 1999; Belousov 1998). Nine species, namely seven ones of *Anillidius* Jeannel, 1928 and by single species of *Pontodytes* Casale & Giachino, 1989 and *Troglocimmerites* Ljovuschkin, 1970, belong to the *Nannotrechus* complex. The *Neotrechus* lineage includes two species of the monotypic genera *Kosswigia* Jeannel, 1947 and *Sbordoniella* Vigna Taglianti, 1980. Finally, the phyletic line of *Duvalius* counts eight species in Anatolia. On the other hand, it is oddly enough that no representative of the former *Aphaenops* series (sensu Jeannel 1928; Casale and Laneyrie 1983) was found in Asia Minor up to now (see also Belousov and Koval 2009: 164), since many taxa of it inhabit the adjacent Balkan Peninsula, Crimea and the Caucasus Major. Recently, authors published evidences that this phyletic series represents a polyphyletic assemblage of genera not related to each other (Faille et al. 2010; Faille et al. 2011). The series is now restricted only to the “Pyrenean clade”, including *Aphaenops* Bonvouloir, 1862, *Hydraphaenops* Jeannel, 1926 and *Geotrechus* Jeannel, 1919. Some other aphaenopsoid genera have no close affinity with this clade being probably offshoots of other lineages. For example, *Doderotrechus* Vigna Taglianti, 1968 seems to be a derivative of the line of *Duvalius* Delarouzée, 1859, whereas *Apoduvalius* and *Speotrechus* belong perhaps to two or more independent lineages related to *Trechus* Clairville, 1806. Unpublished studies on some Alpine and Dinaric subterranean genera, previously placed within the *Aphaenops* series, concluded they may have different origin (Casale A, Faille A, personal communication). According to Casale and Jalžić (1999: 141), *Croatotrechus* Casale & Jalžić, 1999 has no close relationships with other aphaenopsoid genera, and may be it is a sprout of the *Trechus pulchellus* species group. Using the methods of the classic taxonomy, Monguzzi (2011) arrived to the conclusion that *Allegrettia* Jeannel, 1928, together with *Italaphaenops* Ghidini, 1964, is closer to the phyletical line of *Duvalius* than to that of *Aphaenops*.

In the course of a Bulgarian-Turkish cave expedition in the mount of Küre Dağlari during the summer of 2008, my colleague and former director of the National Museum of Natural History Sofia (NMNHS), Dr. Petar Beron was able to collected small series of cave-dwelling blind trechines. After a careful examination, it became evident that this sample should be treated as representing a new species belonging to unknown genus, which combination of characters places it best within a distinct line probably close to a few Balkan taxa of the former *Aphaenops* series.

The discovery of this remarkable species is of an exceptional interest for both the trechine taxonomy and biogeography of the Ponto-Mediterranean region. Hence, the purpose of this report is to introduce this important discovery into science. The new trechine beetle is below described and illustrated, and its systematic position is discussed.

### Material and methods

Prior to be dissected, a photograph of the holotype and drawings of the labrum, clypeus, pronotum, elytra, and protarsomeres were taken using stereoscopic microscope Olympus SZ 60. Afterwards, another specimen was dissected as mouthparts and genitalia were removed, placed into glycerin on a glass slide and outlined using stereoscopic microscope Carl Zeiss Jena Technival 2. At the end, the dissected body parts and the female genital structures were placed in euparal on the same label where a female paratype is glued. The male genitalia were put in a plastic microvial with glycerin for permanent storage pinned to beneath the holotype specimen from whom they were extracted. All the measurements were made mounted on an Olympus SZ 60 stereoscopic microscope.

In general, the morphological terminology follows Jeannel (1926, 1928). Looking for the systematic position of the new genus, we use states of characters traits of great importance, which are often used as generic features in the systematic of Trechini (Jeannel ibid., Casale and Laneyrie 1983). The terms “setae” and “microsetae” (without quotation marks) are used instead “macrochaetes” and “microchaetes” (Quéinnec 2008, Lohaj and Lakota 2010). The term “aphaenopsoid” indicates taxa formerly referred to the *Aphaenops* phyletic line (sensu Jeannel 1928; Casale and Laneyrie 1983). Abbreviations: MHNG (Muséum d’histoire naturelle, Genève, Switzerland); NMNHS (National Museum of Natural History Sofia).

## Taxonomic part

### Beronaphaenops gen. n.

urn:lsid:zoobank.org:act:A1779316-E638-4849-BDA0-A01A8C97B321

http://species-id.net/wiki/Beronaphaenops

**Figure 1.**
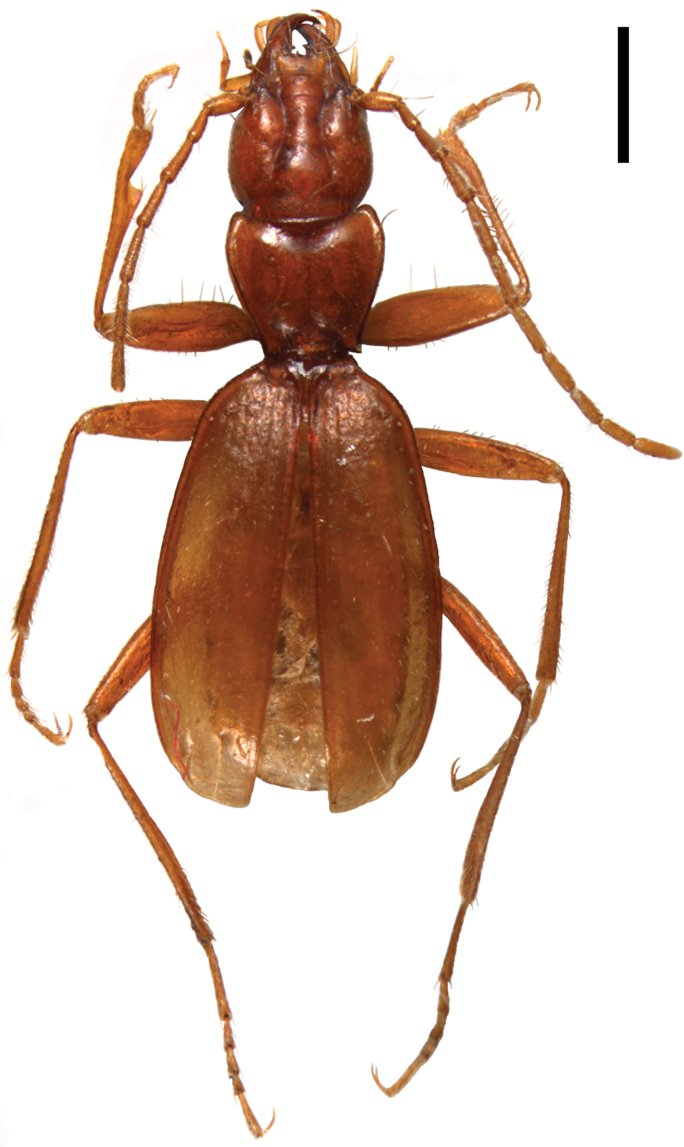
*Beronaphaenops* gen. n. *paphlagonicus* sp. n. Photo of habitus, holotype. Scale bar: 1 mm.

Figs 1 [Fig F2] [Fig F3] [Fig F4] 17

#### Type species.

*Beronaphaenops paphlagonicus* Guéorguiev sp. n., by monotypy

#### Diagnosis.

An anophthalmous trechine genus of uncertain affinity, possessing morphological features in common with the representatives of the former *Aphaenops* line, such as: eyes completely atrophied; front of head with 2-3 supraorbital setae from each side; apical recurrent stria joining stria 5; preapical pore of apical triangle situated on the apical anastomosis of striae 2 and 3; umbilicate series of elytra aggregate or not; protibia entirely pubescent, without longitudinal groove on the external face; male protarsi with first two tarsomeres dilated; aedeagus with inner sac (endophallus) armed with an asymmetrical copulatory piece and/or sclerotised scales in an “anisotopic” position (i.e. displaced laterally within the aedeagus).

The new taxon is unique among all hitherto known genera of Trechini in the following set of characters: integument pubescent; retinacle of left mandible bidentate, retinacle of right mandible tridentate; two pair of supraorbital setae; frontal grooves complete; labium fused; mentum tooth porrect, rather slanting ventrally, deeply bifid at the tip; submentum with row of six prebasilar setae; paraglossae short and fragile, hardly surpassing the anterior margin of ligula; pronotum without basolateral setae at the posterior angles; single discal setigerous puncture in stria 3, situated at the level of fourth pore of humeral umbilicate series; apical triangle of pores complete, preapical pore larger and rather removed from other two pores, external apical pore situated inwards, not close to the recurrent stria; umbilicate series of elytra not aggregate, all but two pores (ones 2 and 8) situated inwards, at discal, not marginal position; humeral umbilicate group consisting of four setigerous pores, as first one situated just before the level of second pore; protibia entirely pubescent, without longitudinal external groove; aedeagus with straight apex, poorly differentiated basal bulb, sagittal carina, and an “anisotopic”, well differentiated copulatory piece surrounded of a field of sclerous scales; apical stylomere of the ovipositor shortened, with basal part unusually broadened and two rather large ensiform setae on tergal position.

The new taxon is also readily distinct from all hitherto known Anatolian genera of Trechini including eyeless species, i.e. *Anillidius*, *Duvalius*, *Koswigia*, *Pontodytes*, *Sbordoniella*, *Troglocimmerites*, in both the pronotum without basolateral setae at the posterior angles and elytra with single discal setigerous puncture in stria 3.

#### Description.

Body elongate, head and pronotum narrow, hind body oval, clearly convex on the dorsum (Fig 1). Integument thin, translucent (as being depigmented), moderately shiny; short, sparse and uniform pubescence presents on both the dorsal and ventral surfaces, tibiae and tarsi densely pubescent. Microsculpture distinctly impressed on the head and elytra, consisting of isodiametric and transverse meshes on head and isodiametric meshes on the elytra, almost indistinct on the pronotum. Colour from reddish-brown on the head and pronotum to yellowish on the elytra and appendages; ventral part of the body more or less lighter than the respective parts of dorsum. Appendages long and slender.

**Figures 2–7. F2:**
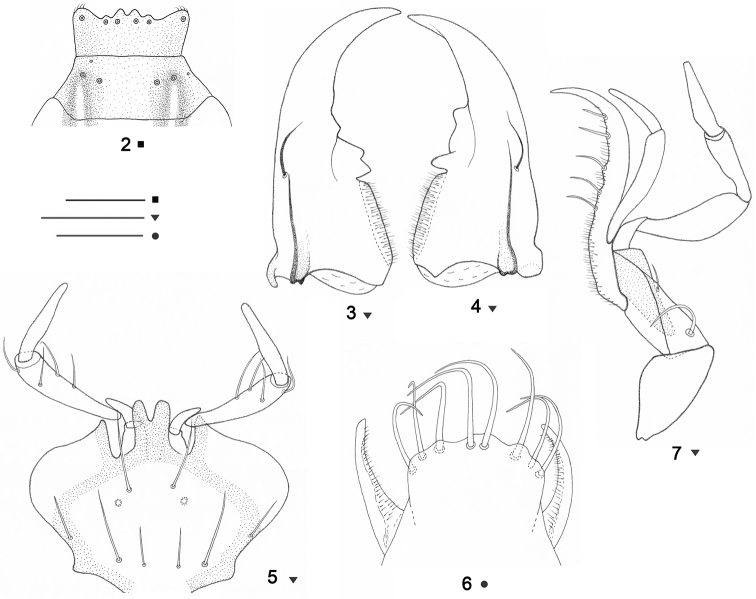
*Beronaphaenops* gen. n. *paphlagonicus* sp. n. **2** Labrum and clypeus in dorsal aspect, holotype. **3** Left mandible in dorsal aspect, paratype ♀ **4** Right mandible in dorsal aspect, paratype ♀**5** Labium in ventral aspect, paratype ♀ **6** Apical part of ligula in ventral aspect, paratype ♀ **7** Left maxilla in ventral aspect, paratype ♀. Scale bar **2, 3, 4, 5, 7**= 0.3 mm; **6**: 0.1 mm.

Head elongate, evidently longer than wide, strongly constricted at the neck; frontal grooves complete, extending onward onto the clypeus, deep along the apical three-fourth of the head where outwardly sinuate, somewhat shallow at the level of posterior supraorbital seta, and again deeper downhill along the collar constriction; eyes perfectly wanting, each one replaced by short, concave, slanting suture; praeocular traits present; genae convex; two pair of supraorbital setae on lines convergent posteriad, the posterior setae being within the frontal grooves. Clypeus sub-hexagonal (Fig. 2), surface covered besides some normal hairs, with four large basic setae, the outer setae being rather longer than the inner ones, one or two additional microsetae situated at marginal apical and lateral positions, as long as the inner basic setae; frontoclypeal sulcus more or less distinct. Labrum transverse (Fig. 2), apically slightly dilated, with six apical setigerous pores, inner and medial pairs of setae situated closer to each other than medial and outer pairs; anterior margin wavy, with a three or five distinct concavities, the middle one much deeper than the outer ones. Mandibles long but fairly stout (Figs 3–4), bidentate, with sharply hooked apices; retinacle of the left mandible bidentate, retinacle of the right mandible tridentate. Labium (Fig. 5) completely fused as no trace of labial suture clear between the mentum and submentum; mentum with pair of setae, median tooth large, long and porrect (at distal position reaching or slightly exceeding the level of epilobes), rather slanting ventrally, deeply bifid at the tip as denticles somewhat divergent onward; submentum with a transverse row of six setae, inner and outer pair of setae shorter than the medial one; ligula slightly broadened apically (Fig. 6), its anterior margin wavy, more pronouncedly in the middle, with eight setae of nearly equal length, two middlemost at ventral position and six lateral at dorsal position, paraglossae short, fragile, transparent, slightly curved and covered with microtrichia dorsolaterally, hardly surpassing the anterior margin of ligula; labial palpus slender (Fig. 5), with penultimate segment quadrisetose, gradually dilated towards the apex, about one-third as long as ultimate segment. Maxillae long and fine (Fig. 7), each with arcuate and sharply pointed lacinia, inner margin of lacinia with 5-6 stout setae at distal position and numerous smaller hairs at medial and proximal position; maxillary palpus entirely glabrous and asetose, slender, ultimate segment fusiform, as long as penultimate one; stipes with two setae at lateroventral position. Antennae filiform, very long and slender, posteriad not exceeding the middle of elytra.

**Figures 8–12. F3:**
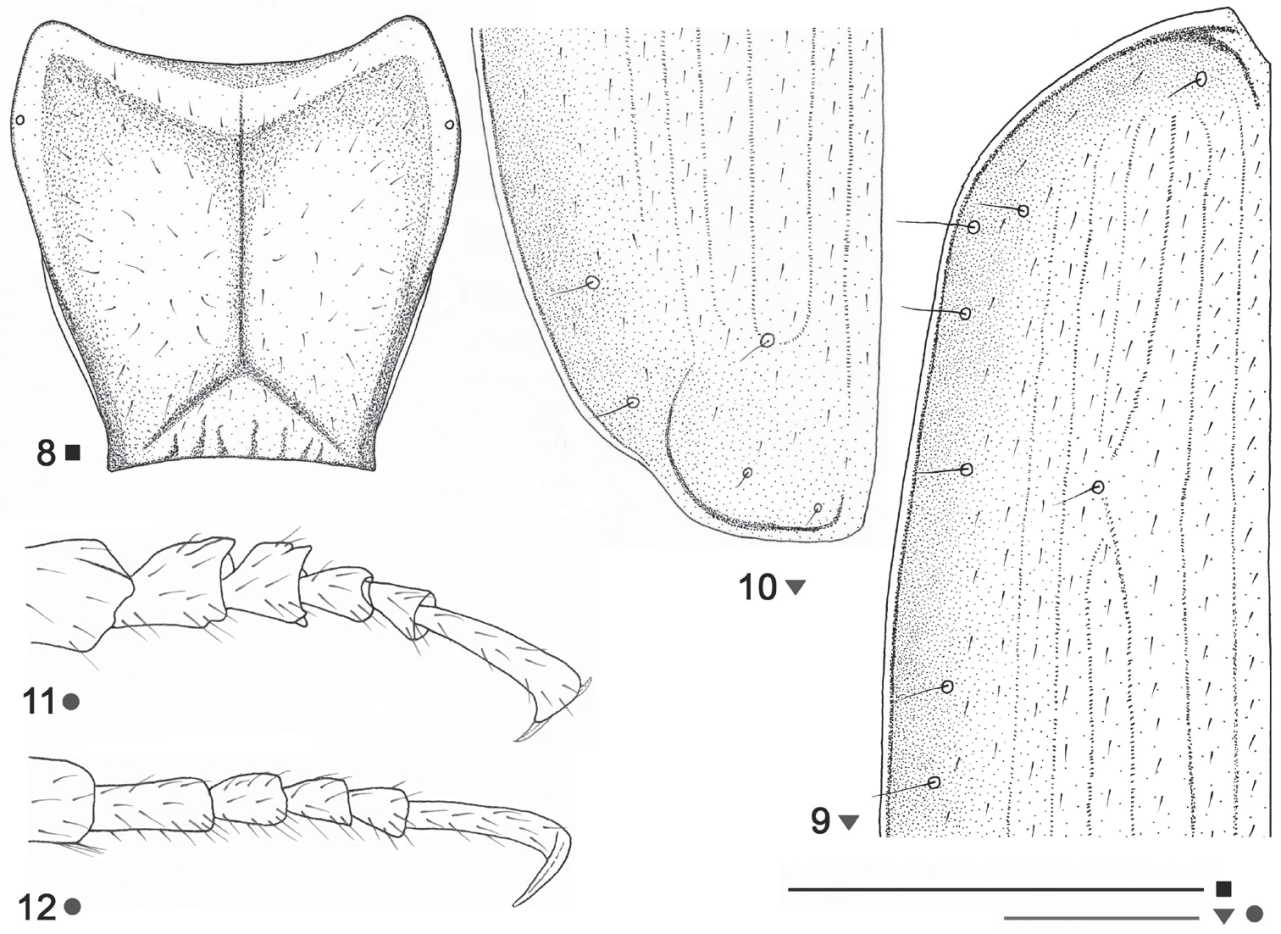
*Beronaphaenops* gen. n. *paphlagonicus* sp. n. **8** Pronotum, holotype **9** Anterior part of left elytron, paratype ♀ **10** Posterior part of left elytron, paratype ♀ **11** Male protarsus in dorsal aspect, paratype ♂ **12** Female protarsus in dorsolateral aspect, paratype ♀. Scale bar **8**: 1 mm; **9, 10**: 0.5 mm; **11, 12**: 0.3mm.

Pronotum cordate (Fig. 8), short in relation with the elytra, wider than long, constricted towards the base, with maximum width at its second-fifth. Anterior margin concave, longer than the posterior one, fore angles prominent, round; posterior margin concave. Sides convex, round anteriad, straight, convergent posteriorly, hardly sinuate before the hind angles; hind angles pointed, projecting outside and turned upwards; lateral margins grooved and their extremities reflexed upward throughout. Anterolateral setae present, posterolateral ones absent. Disc smooth, slightly convex, midline well impressed, not reaching both the basal and apical margins; apical transverse impression less distinct, laterally reaching the anterior margin; basal transverse impression sharply impressed, laterally merging into the basal foveae. Scutellum small, triangular.

Elytra (Figs 1, 9–10) subovate, with shoulders obtuse though still marked, basal border absent. Sides narrower at the basis, slanting at shoulders, becoming wider posteriorly, with maximal width at the third-fourth, round at apex; lateral grooves moderately reflexed upward, and becoming flat at the apical sixth. Disc convex; striae superficial and more or less obliterated at the sides and apically, striae 2 and 3 forming an apical anastomosis, stria 8 fragmentary, not deepening posteriorly; scutellar stria absent; apical recurrent stria well-defined, subparallel to the suture, joining stria 5; intervals flat, each one with single longitudinal row of hairs in the middle; stria 3 with single (? anterior) discal setigerous puncture before the middle, at the level of the fourth pore of humeral umbilicate series; apical triangle of pores complete, the preapical pore as developed as the discal one, on the apical anastomosis of striae 2 and 3, more distant from the apex than from the suture, larger and rather removed from other two apical pores, the internal apical pore somewhat closer to the apex than to the suture, the external apical pore lies inward, at the recurrent stria field, not near to the recurrent stria, closer to the apex than to the suture. Umbilicate series of elytra consisting of 8 pores, not aggregate, very peculiar in disposition, six pores situated inwards, at discal, not marginal position; humeral umbilicate series consisting of four setigerous pores, the first one distant from the marginal gutter, slightly before the level of the second pore, which laying at the marginal gutter, the third pore slightly remote from the gutter, the fourth pore widely distant from both the other pores of humeral group and the gutter, the distance between third and fourth being larger than that between the first and third; two pairs of pores each of the middle and apical groups of umbilicate series not closely set together, the distance between the pores of each group being smaller than the distance between the sixth and seventh, the distance between the pores of the middle umbilicate series almost twice smaller than the distance between the pores of the apical umbilicate series; fifth pore more remote inward than sixth one, seventh pore rather remote inward, the eight pore near to the marginal gutter. Wings absent.

Thorax and lateral sides of abdominal sternites smooth, middle parts of the sternites pubescent, sternites 3-5 each with pair of setigerous punctures; anal sternites with a pair of setae in the males, with two pair of setae in the females.

Legs long and slender; protibia straight, densely pubescent, without longitudinal groove on the external face; tarsi thin, densely pubescent, without modified appendages underneath; protarsi with segments 1 and 2 strongly dilated and inwardly denticulate (more in the first segment, less in the second one), furnished beneath with sexual adhesive appendages in the males (Fig. 11), same segments in the females without such characterization (Fig. 12), segment 1 of the protarsi shorter than segments 2-4 together and clearly shorter than segment 5 in the males, while segments 1 of protarsi hardly shorter than segment 5 in females; both the mesotarsi and metatarsi with segment 1 almost as long as segments 2-4 together and rather longer than segment 5 in the two sexes.

**Figures 13–16. F4:**
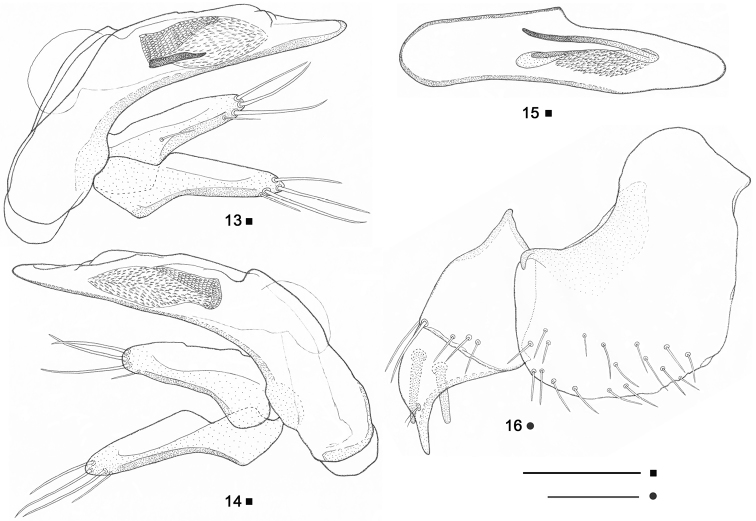
**13** *Beronaphaenops* gen. n. *paphlagonicus* sp. n. Aedeagus in left lateral aspect, holotype. **14** Aedeagus in right lateral aspect, holotype **15** Aedeagus in dorsal aspect, holotype**16** Left ovipositor in ventral aspect, paratype ♀. Scale bar **13, 14, 15**: 0.2 mm; **16**: 0.1 mm.

Aedeagus (Figs 13–15) with straight apex and poorly differentiated basal bulb in lateral aspect, thinner to the apex, moderately wide at basal and medial parts; sagittal carina moderately large, round; ventral margin straight apically, slightly concave at the middle; dorsal margin little convex with preputial bulge before the basal part; apical orifice very large, reaching the basal part of aedeagus; basal orifice small, flat, weakly differentiated; aedeagus slightly asymmetrical in dorsal aspect, with left side concave at the middle, apex widely round. Inner sac (endophallus) with an “anisotopic”, well differentiated, large copulatory piece surrounded of a field of sclerous scales, situated in the medial and distal parts of the aedeagus; copulatory piece double, its ventral branch partially visible at left lateral aspect, same longer in dorsal aspect than in the lateral one, V-shaped, with dorsal branch well visible from above, more chitinized proximally, ventral branch less evident as sunken in compact chitin. Parameres elongate and thick, each one with four apical setae, the distal three large and long, the proximal one smaller, left paramere with additional minute seta in medial position.

Ovipositor consisting of large valvifer and two-segmented stylus (Fig. 16). Valvifer nearly twice larger than the stylus, with more chitinized, glabrous internal margin and less chitinized, pubescent (17-20 setae visible) external margin in sternal position. Basal stylomere of stylus larger than the apical one, with three marginal setae at central sternal position and two bigger ones distally, one on the sternal face, another on the tergal face; apical stylomere subtriangular, with two rather large ensiform setae (as long as or longer than the half length of stylomere) on tergal position, and sensorial fovea bearing two minute nematiform setae on sternal position.

#### Etymology.

The generic epithet is a compound word, based on the family name of the collector, Dr. Petar Beron, remarkable Bulgarian biospeleologist and discoverer of numerous troglobiont invertebrates new for the science, and *Aphaenops* (meaning “without visible eyes”). It is treated as a Latin masculine.

#### Affinities.

In order to look for the systematic position of the new genus, the last is compared with different genera of blind Trechini from the Anatolian Peninsula and adjacent areas of the Balkan, Crimea, and Caucasus.

For the semi-aphaenopsoid shape of body and first two protarsomeres dilated in the males, *Beronaphaenops* gen. n. *paphlagonicus* sp. n. superficially resembles taxa of *Duvalius*, but the former is markedly distinct from the latter in: the integument of body covered by short and sparse pubescence; labium completely fused; pronotum without posterolateral setae at the hind angles; single discal pore in elytral stria 3; humeral group of umbilicate series of elytra not aggregate; “anisotopic” position of the copulatory piece of aedeagus.

The new genus is also easily distinct from the representatives of the *Nannotrechus* complex (sensu Belousov 1998) in the following series of characters: front, temples and vertex of head without parietal and temporal microsetae; labium completely fused; pronotum without posterolateral setae at the hind angles; single discal pore in elytral stria 3; humeral group of umbilicate series not aggregate; male protarsus with first two segments dilate. Hence, a relationship between the new taxon and representatives of this Caucasian lineage is almost unlikely.

The Anatolian genera *Kosswigia* and *Sbordoniella*, related each other (Casale and Vigna Taglianti 1999), belong to the *Neotrechus* series (sensu Casale and Laneyrie 1983). They share some traits with *Beronaphaenops* gen. n.: labium completely fused; submentum with row of 6 setae; humeral group of umbilicate series of elytra not aggregate, as distance between the humeral umbilicate pores 3 and 4 nearly twice longer than that between pores 2 and 3; protibia entirely pubescent, without longitudinal groove on the external face; “anisotopic” position of the copulatory piece of aedeagus. However, the new taxon differs from its Anatolian neighbors in: the specific structure of the mentum, including deeply bifid tooth; pronotum without posterolateral setae at the hind angles; single discal pores in elytral stria 3; male protarsus with first two segments dilated; segment 4 of protarsi and mesotarsi without ventral modification (the state of this character unknown in *Sbordoniella*). At present, it is not clear if this partial resemblance is a result of common origin or of convergent evolution. In spite of the geographical nearness, the listed differences display a significant phyletic diversion between the species from the Küre Dağlari Mt. and those from the Western Taurus.

Regarding the Balkan eyeless Trechini, there are genera from two lineages, which may have remote affinity with the remarkable beetle from Turkey. Among the other groups of the *Neotrechus* series, an essential affinity of the East Alpine genus *Orotrechus* J. Müller, 1913 with the new genus makes impression. Both genera share the following states: labium fused; mentum tooth bifid; pronotum without posterolateral setae at the hind angles (but a reduced posterolateral seta is found in some species from the former genus); apical triangle of pores complete, with external apical pore migrated inwards (i.e. situated at the recurrent stria field, not close to the recurrent stria); umbilicate series of elytra not aggregate; protibia entirely pubescent, without longitudinal external groove; “anisotopic” position of the copulatory piece of aedeagus. Despite all, the new genus is distinct from *Orotrechus* in: single discal pores in elytral stria 3; male protarsus with first two segments dilated; number of the apical setae on parameres. It is very difficult to say, if the explained above similarity is a result of common origin. The lost of the setae in elytral stria 3 is an apotypy in *Beronaphaenops* gen. n. However, being a simple morphological regression, this trait is of low phyletic weight. The number of dilated segments in the male protarsi may be also of little importance, since it varies within the limits of one and same lineage of Trechini (Casale and Giachino 1989, Belousov and Zamotajlov 1997). For instance, within the *Nannotrechus* complex, only the monotypic genus *Pontodytes* has two first dilated segments in the males, while all other genera have first segment dilated. As well, the following transition is known among the Caucasian aphaenopsoid genera: male protarsus with 2 dilated segments (*Caucasorites shchurovi* Belousov & Zamotajlov, 1997 and *Caucasorites victori* Belousov, 1999) - same with two hardly dilated segments (*Caucasorites kovali* Belousov 1999) - same with 2 not dilated segments (all species of *Jeannelius* Kurnakov 1959, Belousov 1999).

The new genus and all the eleven Balkan aphaenopsoid genera known till now (Lohaj and Lakota 2010, Casale et al. 2012) share four important features (not enumerated below when listing similarities!): head with 2 (3) supraorbital setae from each side; apical recurrent stria joining stria 5; protibia entirely pubescent, without longitudinal groove on its external face (protibia sulcate only in *Albanotrechus* Casale & V.B. Guéorguiev, 1994); male protarsus with first two segments dilated. In addition, *Beronaphaenops* gen. n. shares with the most of these genera: pronotum without posterolateral setae at the hind angles and “anisotopic” conformation of the copulatory piece of aedeagus.

Among the Balkan genera of this ensemble, *Acheroniotes* Lohaj & Lakota, 2010, *Croatotrechus* Casale & Jalžić, 1999, and *Jalzicaphaenops* Lohaj & Lakota, 2010 together are remarkable for: their stalklike body; glabrous elytra with effaced humeri; submentum with row of 2-4 prebasilar setae; pronotum with posterolateral setae at the hind angles. Each of these Balkan taxa possesses a set of features, which additionally reveal the lack of relationships between them and the genus from Anatolia. *Acheroniotes* differs from the new taxon in: the frontal grooves incomplete; labium imperfectly fused; mentum without median teeth; four-five discal setae in elytral stria 3, as well additional microsetae in striae 3 and 5; humeral group of umbilicate series aggregated. *Croatotrechus* is remarkable for: the two discal setae in elytral stria 3; humeral umbilicate series with pore 4 situated at the middle of elytron length; segment 4 of the mesotarsi with long ventral appendage. *Jalzicaphaenops* is distinct from the new genus in: the labium imperfectly fused; mentum without median teeth; pronotum with a pair of setae on the disc near midline; two discal setae in elytral stria 3; humeral group of umbilicate series aggregated.

According to Casale et al. (2012), the North Dinaric genera *Dalmataphaenops* Monguzzi, 1993 and *Velebitaphaenops* Casale & Jalžić, 2012 may have common derivation. Both genera share some important traits: large size (10–14 mm); head and elytra glabrous; incomplete frontal grooves; submentum with row of 8–10 basilar setae; labium not fused; mentum tooth wide, slightly prominent, moderately bifid; humeral group of the umbilicate series aggregate; similar structure of the inner sac of aedeagus. These states are different or their polarity is opposite in *Beronaphaenops* gen. n. which exclude eventual relationships.

The genera *Minosaphanops* Quéinnec, 2008 and *Scotoplanetes* Absolon, 1913 are also stated as related each other (Quéinnec 2008: 160–161). These genera share some characters, which however are different in the new genus: stalklike body; mentum without median tooth; four or more discal setae in elytral stria 3; umbilicate series of elytra aggregate; reduced inward denticulation of segments 1–2 in the male. In addition, *Minosaphanops* has incomplete frontal grooves, labium imperfectly fused, and elytra with microsetae in striae 2 and 5, while *Scotoplanetes* possesses 3-6 discal setae in stria 5 and a rather different conformation of the aedeagus. Based on these facts, we do not suppose phyletic nearness between these two Balkan genera and *Beronaphaenops* gen. n.

*Derossiella* Quéinnec, 2008 differs from the new genus in: the stalklike shape of the body; completely glabrous integument; labium not fused; mentum tooth simple; umbilicate series of elytra not typically aggregated, as only pore 7 distant from marginal gutter (also, pore 1 of the humeral group set isolated from the other pores, while pores 2–4 equidistant); two discal setae on the site of elytral stria 3, and presence of two microsetae between the discal setae.

We exclude also direct affinity of the new taxon with *Adriaphaenops* Noesske, 1928, which include species with: stalklike body; incomplete, very short frontal grooves; teeth of right mandible reduced or vanished; labium not fused; mentum tooth simple; supraorbital setae more or less reduced; two discal setae in elytral stria 3. However, this genus partakes with the new genus: integument entirely pubescent; pronotum without posterolateral setae at the hind angles; umbilicate series of elytra not aggregated; “anisotopic” conformation of the copulatory piece of aedeagus.

*Aphaenopsis* J. Müller, 1913 is distinct from *Beronaphaenops* gen. n. in: the glabrous integument of body; incomplete frontal grooves; labium not fused; mentum tooth simple; ligula with prominent anterior process, which bears single long seta; elytra with two discal pores in stria 3; internal apical pore missing. The similarities between both genera are: submentum with 6 prebasilar setae; ligula with short and fragile paraglossae; pronotum without posterolateral setae at the hind angles; rather similar conformation of the umbilicate series of elytra (namely the disposition of the humeral pores 1-4 and similar distance between the pores of middle and apical groups); “anisotopic” conformation of the copulatory piece of aedeagus.

*Albanotrechus* Casale & V.B. Guéorguiev, 1994 differs from the new genus in: the glabrous integument (but sparse pubescence present on the temporae, abdominal sternites, and external intervals of elytra in the former); labium not fused; elytra with two discal pores in stria 3, as anterior one at the level of umbilicate pores 1-2, and posterior one just before the level of pore 5; external apical pore of the apical triangle near to apical recurrent stria; protibiae grooved and rugose on the external face; elongate aedeagus with apical lamella curved upward; apical stylomere with three moderately large ensiform setae on tergal position. On the contrary, the two genera share some important similarities: complete frontal grooves; mentum tooth bifid (moderately prominent in *Albanotrechus*, porrect in the new genus); submentum with 6 prebasilar setae; ligula with 8 setae on its anterior margin; paraglossae fragile and relatively short; pronotum without posterolateral setae at the hind angles; elytral shoulders not effaced; markedly similar conformation of the umbilicate series of elytra, especially the structure of the humeral umbilicate group (e.g.: pore 1 rather remote inwards, situated at or slightly before the level of second pore, pore 2 laying almost at the marginal gutter, pores 3 and 4 clearly remote from gutter, the distance between pores 3 and 4 larger than the distance between pores 2 and 3) and pore 8 situated near to the marginal gutter; “anisotopic” conformation of the copulatory piece of aedeagus. To all appearances, among the Balkan aphaenopsoid taxa, the new taxon shares most common traits with *Albanotrechus*. However, the phyletic distance between these two genera is obvious.

One Crimean and one Caucasian genus, respectively *Pseudaphaenops* Winkler, 1912 and *Meganophthalmus* Kurnakov, 1959, both postulated to be related each other (Jeannel 1960, Belousov and Koval 2009), share with the new taxon only a few features of importance, which exclude any relationships. *Pseudaphaenops* and *Beronaphaenops* gen. n. have male protarsus with first two segments dilate and protibiae pubescent and ungrooved on external face. These genera differ in the state of many important characters: structure of the labium (fused or not); number of setae on the submentum (6 or 8); integument of the elytra (pubescent or glabrous); state presence/absence of posterolateral setae on the pronotum; number of discal setae on elytra; aggregation of the humeral group of umbilicate series. *Beronaphaenops* gen. n. *paphlagonicus* sp. n. strongly differs from the species of *Meganophthalmus* Kurnakov, 1959 in: the entirely pubescent body (vs. pilosity available only on the anterior surface of protarsi in *Meganophthalmus* spp.); sub-triangular pronotum (vs. elongate pronotum); sub-ovoid shape of the elytra with maximal width posteriad (vs. ovoid shape of the elytra with maximal width anteriad, or at least in the middle as in *Meganophthalmus kravezi* Komarov, 1993); mentum tooth bifid (vs. mentum tooth simple); labium fused (not fused in *Meganophthalmus* spp.); number of the discal setae on elytra; humeral group of umbilicate series of elytra not aggregate; segment 4 of the protarsi and mesotarsi not modified ventrally (vs. segment 4 of the protarsi and mesotarsi with hyaline appendage ventrally).

The differences between the monotypic genus *Taniatrechus* Belousov & Dolzhanski, 1994, which has been postulated to be related to the Balkan *Pheggomisetes* Knirsch, 1923 (Belousov and Koval 2009), and the new genus are more than the traits they share. The Caucasian taxon is distinct from the Anatolian one in: the body entirely glabrous (excl. protibiae); temporae of head from each side with 6-10 supraorbital setae situated on the external side of frontal groove, laterad and on the low surface of genae; labium not fused; mentum tooth simple, hardly prominent or missing; submentum with a transverse row of 9-10 setae; pronotum very long and parallel-sided as posterolateral pores present; elytra with two discal setae in stria 3; segment 4 of protarsi and mesotarsi with hyaline appendage ventrally.

The genus *Jeannelius* Kurnakov, 1959 includes four species with first two segments of the protarsi not dilated in the males. This genus and *Beronaphaenops* gen. n. share only two similarities: mentum tooth bifid and submentum with 6 prebasilar setae. The differences between these taxa are more than the similarities. All species of the Caucasian genus share features, which clearly distinguish them from the new species: rather big species (7.5–10.2 mm; vs. 5.7–6 mm in *Beronaphaenops* gen. n. *paphlagonicus* sp. n.); entirely glabrous integument (excl. temples and protibia); labium not fused; humeral group of the umbilicate series of elytra aggregate (only first umbilicate pore placed on stria 7th); male protarsi with first two tarsomeres not dilated; segment 4 of the protarsi and mesotarsi with hyaline appendage ventrally.

The most recently described aphaenopsoid groups from the Caucasus are *Caucasorites* Belousov & Zamotajlov, 1997 and *Caucasaphaenops* Belousov, 1999, which for the moment seem more related to each other than each of them is to another genus of Trechini (Belousov 1999). Virtually, these genera and the new one do not share any important trait. To some extent, they resemble each other in the integument entirely pubescent. But, the rate of pilosity is different: the Caucasian species are densely pubescent, while the one from the Küre Dağlari Mt. is only sparsely pubescent. There are more differences between the two Caucasian genera and *Beronaphaenops* gen. n. The latter differs from the formers in: the head without parietal and temporal microsetae (vs. head with parietal or/and temporal microsetae distinct from the surrounding ordinary hairs); labium completely fused (vs. labium not fused); mentum tooth porrect, deeply bifid (vs. mentum tooth not projecting, simple or cleft at the apex); submentum with transverse row of 6 basic setae, without additional microsetae (vs. submentum with transverse row of 6-8 basilar setae and 4–5 additional microsetae); hind angles of the pronotum without basolateral setae (vs. pronotum with basolateral setae); elytra with single discal pore in stria 3, posterior one wanting (vs. elytra with two discal pores in stria 3 in *Caucasorites kovali* Belousov, 1999 and *Caucasaphaenops molchanovi* Belousov, 1999, or elytra with single, posterior discal pore in stria 3 in *Caucasorites shchurovi* Belousov & Zamotajlov, 1997 and *Caucasorites victori* Belousov, 1999); humeral group of the umbilicate series of elytra not aggregate (vs. humeral group of umbilicate series of elytra aggregate); segment 4 of the protarsi and mesotarsi without appendage ventrally (vs. segment 4 of the protarsi and mesotarsi with hyaline appendage ventrally).

Taking into consideration the weight of more important characters, we should note that *Beronaphaenops* gen. n. *paphlagonicus* sp. n. seems unrelated to any of the present trechine taxa from both the Crimean Peninsula and the Caucasus Major. All the aphaenopsoid genera from the last two areas have labium not fused, umbilicate series of elytra aggregate (only *Jeannelius* and *Taniantrechus* with umbilicate pore 1 positioned in stria 7), and segment 4 of the protarsi and mesotarsi with hyaline appendage ventrally.

There are at least five probable apotypies found in *Beronaphaenops* gen. n., e.g.: mentum tooth large, long and porrect (at distal position reaching or slightly exceeding the level of epilobes), rather slanting ventrally, deeply bifid at the tip (i); short and fragile paraglossae, hardly surpassing the anterior margin of ligula (ii); absence of posterolateral seta of the pronotum (iii); absence of posterior discal pore in elytral stria 3 (iv); apical stylomere of the ovipositor shortened, with basal part unusually broadened (v). These states are listed here, because they could be used eventually in a further quest for phylogenetic inferences or cladistic analysis.

In conclusion, the discussion in this section, as well as, a very peculiar diagnostic combination (see section Diagnosis) seem to us enough to place the new genus at the best after the Balkan taxa of the former *Aphaenops* series and before the *Neotrechus* series of Trechini. From among the peri-Pontic aphaenopsoid Trechini, the new genus shares most important features with *Albanotrechus*. On the other side, there is also a striking affinity with the East Alpine genus *Orotrechus*, which belong to the *Neotrechus* series. In any case, a very ancient origin of *Beronaphaenops* gen. n. from a “western” or North Aegeidean lineage could be possible. Actually, for the time being, the affinities of the new genus are not clear, and this is probably due to the numerous convergent characters shared by hypogean Trechini.

### Beronaphaenops paphlagonicus sp. n.

urn:lsid:zoobank.org:act:E9DD4767-61C1-4347-BE9A-E505D072B8C3

http://species-id.net/wiki/Beronaphaenops_paphlagonicus

#### Type material.

Holotype ♂, labelled: “Turkey, Pinarbasi Distr., Milli Park Kűre Dağlari, cave Eşek Çukuru Mağarası 2, 14.VII.2008, P. Beron leg.” [typeset] / “Holotype *Beronaphaenops* gen. n. *paphlagonicus* sp. n. B. Guéorguiev det. 2011” [red typeset] (NMNHS); paratypes 1 ♂, 3 ♀♀, labelled: “Turkey, Pinarbasi Distr., Milli Park Kűre Dağlari, cave Eşek Çukuru Mağarası 2, 14.VII.2008, P. Beron leg.” [typeset] / “Paratype *Beronaphaenops* gen. n. *paphlagonicus* sp. n. B. Guéorguiev det. 2011” [red typeset] (MHNG; NMNHS).

#### Diagnosis.

A medium sized trechine, included in *Beronaphaenops* gen. n., with pubescent integument, elongated head, complete frontal grooves, labrum serrate on the anterior margin, labium fused, mentum tooth large, long, and deeply bifid at the tip, pronotum short and narrow, without posterolateral setae, and with pointed hind angles, elytra with superficial striae, one discal pore in stria 3, umbilicate series not aggregate, aedeagus with straight apex and poorly differentiated basal bulb, apical stylomere short, broadened basally, with two large ensiform setae on tergal position.

#### Description.

Medium sized, overall length (from the apex of longer mandible to the apices of elytra): 5.72–5.95 (mean 5.87) mm; maximum width: 1.88–1.98 (mean 1.91) mm. General shape as in Fig. 1. Integument thin, translucent, covered by short, rather sparse and uniform pubescence, tibiae and tarsi densely pubescent. Microsculpture distinct on the head and elytra, very fine, almost indistinct on the pronotum, consisting of isodiametric and transverse meshes on the head and isodiametric ones on the elytra. Colour reddish-brown on the head and pronotum to yellowish on the elytra and appendages.

Head rather elongate, 1.49–1.59 (mean 1.56) times as long as wide, 1.6–1.74 (mean 1.68) times as long as the pronotum, slightly narrow than the pronotum: 0.88-0.93 (mean 0.91) times as wide as pronotum, clearly constricted at the neck; frontal grooves complete, extending onward onto the clypeus, deep along the apical three-fourth of the head where outwardly sinuate, somewhat shallow at the level of posterior supraorbital seta, and again deeper downhill along the collar constriction; genae convex. Antennae filiform, very long and slender, 0.63-0.65 times as long as overall body length, posteriad not exceeding middle of elytra.

Pronotum cordate, 1.15–1.21 (mean 1.19) times as wide as long, constricted towards the base. Anterior margin 1.24–1.27 times as wide as the posterior margin, 0.78–0.83 times wide as the pronotal maximum width, gradually concave towards the middle, the fore angles prominent, round. Posterior margin 0.63–0.65 times wide as the pronotal maximum width, concave inwards as the hind angles projecting back. Sides convex, round anteriad, straight, convergent posteriorly, hardly sinuate before the acute hind angles, which slightly projecting outside and turned upwards. Lateral margins grooved throughout, less anteriorly, more posteriorly, their extremities reflexed upward throughout, less anteriorly, more posteriorly. Anterolateral setae present, with length exceeding two-thirds of the pronotal side length, situated at the place of maximal pronotum width, posterolateral setae absent. Disc smooth, slightly convex, midline well impressed, not reaching both the basal and apical margins; apical transverse impression less distinct, continuous, laterally reaching the anterior margin, basal transverse impression sharply impressed, continuous, laterally merging into the small, but distinct basal foveae. Scutellum small, triangular.

Elytra subovate, 1.67–1.78 times longer than wide, 3.28–3.55 times as long as the pronotal maximum length, and 1.6–1.72 times wider than the pronotal maximum width. Sides narrow and slanting at the basis, becoming wider posteriorly, with maximal width at the third-fourth, round at the apex; lateral grooves reflexed moderately upward, becoming flat at the apical sixth; shoulders obtuse though still marked, basal border absent. Disc convex; striae superficial, more or less obliterated at the sides and apically, scutellar stria absent, apical recurrent one present; intervals flat, each one with single longitudinal row of hairs in the middle; stria 3 with single discal pore before the middle; apical triangle of pores complete; umbilicate series of elytra consisting of 8 pores, not aggregate.

Legs long and slender; protibia straight, densely pubescent, without longitudinal groove on the external face; tarsi thin, densely pubescent, segments 1 and 2 in the males strongly dilated and inwardly denticulate at the apices.

Male genitalia: as in Figs 13–15 (see also the genus description).

Female genitalia: as in Fig. 16 (see also the genus description).

#### Etymology.

The specific epithet is a noun in the nominative singular in apposition. It honors Paphlagonia, an ancient country on the today’s Kastamonu Province of Turkey.

**Figure 17.**
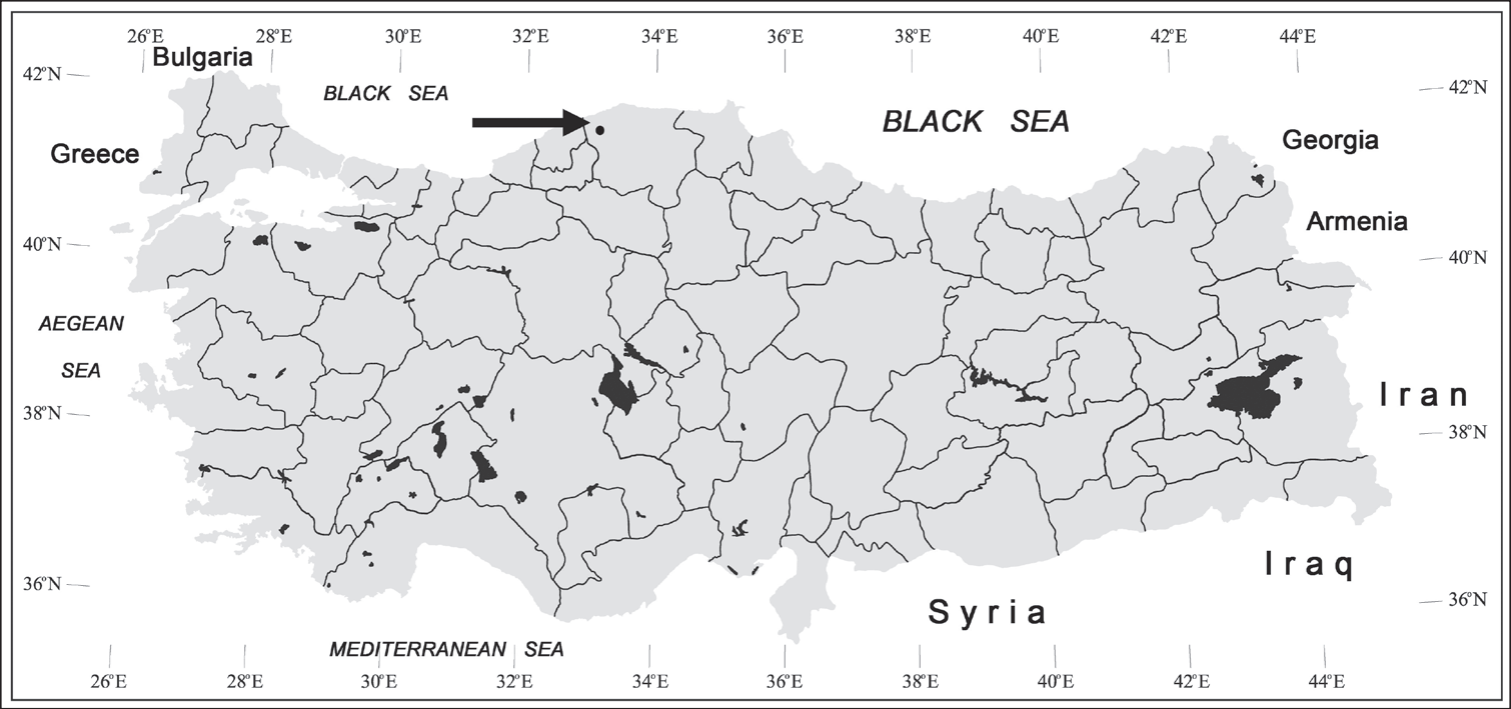
Administrative map of Turkey with black arrow and dot marking approximately the type locality of *Beronaphaenops* gen. n. *paphlagonicus* sp. n.

**Distribution.** Up to now the new species is known only from its type locality, the cave Eşek Çukuru Mağarası 2 (Fig. 17). The cave is situated in the Küre Dağları Milli Parkı, a Turkish national park which includes a forested, well protected karstic area in the Küre Mountain. The most important distinctive feature of this karstic zone is that it harbors old growth natural beech and fir forests, endemic plant species, forest ecosystems rich in biological diversity, as a result of being in the humid climatic zone. The climate in the inner part of the area is harsh.

##### Key to the Anatolian genera of Trechini

**Table:** 

1 (2)	Basal bead of elytra reaching scutellum or stria 1	*Thalassophilus* Wollaston, 1854
2 (1)	Basal bead of elytra lacking or reaching stria 3 at most.
3 (6)	Eyes pilose, well developed with function ommatidia. Penultimate segment of labial palpomeres with more than four setae. Fore tibia with spine apically on outer side.
4 (5)	Protibiae feebly grooved on external face. Larger size (ca. 3.8 mm)	*Neoblemus* Jeannel, 1923
5 (4)	Protibiae ungrooved on external face. Smaller size (2.2-2.9 mm)	*Perileptus* Schaum, 1860
6 (3)	Eyes glabrous, developed or atrophied. Penultimate segment of labial palpomeres with four setae. Fore tibia without spine apically on outer side.
7 (8)	Anterior face of protibiae glabrous	*Trechus* Clairville, 1806
8 (7)	Anterior face of protibiae pubescent, mostly in apical part.
9 (10)	Apical recurrent stria joining stria 3. Pubescent species with well developed eyes. Submentum with a transverse row of 8-12 setae	*Trechoblemus* Ganglbauer, 1892
10 (9)	Apical recurrent stria joining stria 5. Glabrous or pubescent species with rudimentary or absent eyes. Submentum with a transverse row of 6–9 setae.
11 (18)	Humeral group of umbilicate series aggregate as four pores adjoining marginal gutter. Labium not fused (mentum separated by submentum by distinct labial suture).
12 (15)	Head without parietal or/and temporal microsetae.
13 (14)	Pronotum and elytra glabrous. Submentum with a transverse row of 6 setae. Male protarsus with first two tarsomeres dilated. Eight endemic species with scattered distribution in Anatolia throughout, with body size: 3.7–7.5 mm	*Duvalius* Delarouzée, 1859
14 (13)	Pronotum and/or elytra with very short and scattered pubescence. Submentum with a transverse row of 8 or more setae. Male protarsus with only first tarsomere dilated. Seven endemic species with scattered distribution in NW and SW Anatolia, with body size: 2.6–4.2 mm.	*Anillidius* Jeannel, 1928
15 (12)	Head with parietal or/and temporal microsetae.
16 (17)	Mentum tooth single, large and pointed. Head with parietal microsetae, without temporal ones. Male protarsus with first two tarsomeres dilated. One endemic species from Ordu Province, NE Anatolia, with size 3.2 mm	*Pontodytes* Casale & Giachino, 1989
17 (16)	Mentum tooth bifid. Head with both parietal and temporal microsetae. Male protarsus with only first tarsomere dilated. One endemic species from Artvin Province, NE Anatolia, with size: 2.8–3.1 mm	*Troglocimmerites* Ljovuschkin, 1970
18 (11)	Humeral group of umbilicate series not aggregate as some pores situated inwards, more or less distant from marginal gutter. Labium completely fused.
19 (22)	Pronotum with pair of long posterolateral setae situated near hind angles. Male protarsus with only first tarsomere dilated. Mentum tooth simple.
20 (21)	Head and elytra glabrous. Pores 1 and 4 of humeral group of umbilicate series rather distant from marginal gutter, pores 2 and 3 adjoining marginal gutter. Larger size (ca. 6 mm). One endemic species from Konya Province, SW Anatolia.	*Kosswigia* Jeannel, 1947
21 (20)	Head and elytra pubescent. Pores 3 and 4 of humeral group of umbilicate series little distant from marginal gutter, pores 1 and 2 adjoining marginal gutter. Smaller size (3.85–4.2 mm). One endemic species from Antalya Province, SW Anatolia	*Sbordoniella* Vigna Taglianti, 1980
22 (19)	Pronotum without posterolateral setae situated near hind angles. Male protarsus with first two tarsomeres dilated. Mentum tooth bifid. One species from Kastamonu Province, NW Anatolia, with size: 5.7–6 mm	*Beronaphaenops* gen. n.

## Supplementary Material

XML Treatment for Beronaphaenops

XML Treatment for Beronaphaenops paphlagonicus
